# *Rhodnius prolixus*: Identification of missing components of the IMD immune signaling pathway and functional characterization of its role in eliminating bacteria

**DOI:** 10.1371/journal.pone.0214794

**Published:** 2019-04-03

**Authors:** Nicolas Salcedo-Porras, Alessandra Guarneri, Pedro L. Oliveira, Carl Lowenberger

**Affiliations:** 1 Centre for Cell Biology, Development, and Disease, Department of Biological Sciences, Simon Fraser University, Burnaby, British Columbia, Canada; 2 Instituto René Rachou, Avenida Augusto de Lima, Belo Horizonte, Minas Gerais, Brazil; 3 Instituto de Bioquímica Médica, Universidade Federal do Rio de Janeiro, CCS, Ilha do Fundão, Rio de Janeiro, Brazil; Natural Resources Canada, CANADA

## Abstract

The innate immune system in insects is regulated by specific signalling pathways. Most immune related pathways were identified and characterized in holometabolous insects such as *Drosophila melanogaster*, and it was assumed they would be highly conserved in all insects. The hemimetabolous insect, *Rhodnius prolixus*, has served as a model to study basic insect physiology, but also is a major vector of the human parasite, *Trypanosoma cruzi*, that causes 10,000 deaths annually. The publication of the *R*. *prolixus* genome revealed that one of the main immune pathways, the Immune-deficiency pathway (IMD), was incomplete and probably non-functional, an observation shared with other hemimetabolous insects including the pea aphid (*Acyrthosiphon pisum*) and the bedbug (*Cimex lectularius*). It was proposed that the IMD pathway is inactive in *R*. *prolixus* as an adaptation to prevent eliminating beneficial symbiont gut bacteria. We used bioinformatic analyses based on reciprocal BLAST and HMM-profile searches to find orthologs for most of the “missing” elements of the IMD pathway and provide data that these are regulated in response to infection with Gram-negative bacteria. We used RNAi strategies to demonstrate the role of the IMD pathway in regulating the expression of specific antimicrobial peptides (AMPs) in the fat body of *R*. *prolixus*. The data indicate that the IMD pathway is present and active in *R*. *prolixus*, which opens up new avenues of research on *R*. *prolixus-T*. *cruzi* interactions.

## Introduction

The immune system of arthropods relies exclusively on an innate response triggered by the detection of conserved Pathogen Associated Molecular Patterns (PAMPs) found on the surface of microorganisms [1]. In arthropods, the elimination of opportunistic pathogens, the regulation of beneficial symbionts, and the immune response to eukaryotic parasites is orchestrated by a handful of well conserved molecular pathways such as the Toll pathway, the immune deficiency (IMD) pathway, the JAK-STAT pathway, and the RNAi pathway [2–5]. Each of these pathways has been associated, historically, with the control of subgroups of pathogens. Traditionally the Toll pathway has been considered to be induced by Gram-positive bacteria and fungi, the IMD pathway by Gram-negative bacteria, and JAK-STAT and RNAi pathways by viruses, although we now know that there is cross talk among the various pathways [6–9]. Most of our understanding of immune responses has been derived from dipteran models such as *Aedes aegypti* and *Drosophila melanogaster* and it has been assumed that these findings apply universally to all other arthropods. Current evidence from ticks, chelicerates, hemipterans, and lice describe a deviation from the idea of complete conservation of immune responses and pathways, highlighting the need to study basic immune responses in disparate species [9–15].

One of the first organisms used to study basic concepts of insect physiology was the hematophagous triatomine bug, *Rhodnius prolixus* [16–18]. This insect also is a principal vector of *Trypanosoma cruzi*, which causes Chagas disease in humans, killing ~12,000 people annually and affecting 6–10 million people worldwide [19,20]. Triatomines ingest *T*. *cruzi* trypomastigotes when the feed on infected vertebrates. In contrast to other vector borne parasites that invade the insect body cavity and replicate in salivary glands, *T*. *cruzi* develops and replicates solely within the gastrointestinal (GI) tract of the insect, and infective forms of the parasite are released and transmitted with the feces as the insect feeds. If this parasite invades the body cavity of the insect vector it is promptly eliminated by elements of the innate immune system [21,22]. Moreover, *R*. *prolixus* is the only triatomine to have had its genome sequenced and published, which makes it an ideal model to investigate innate immune responses in a non-dipteran blood feeding insect vector.

The molecular interactions between trypanosomes, the vector’s innate immune system, and the obligate endosymbiotic bacterial symbionts of *R*. *prolixus* have received increased attention over the last decade. The principal symbiotic bacterium in the microbiome of *R*. *prolixus* is the Gram-positive bacterium *Rhodococcus rhodnii*, found mainly in the anterior midgut (AM) region of the insect. Within 48 hours of ingesting a blood meal, the population of *R*. *rhodnii* increases 10,000 times [23,24]. Concomitantly, the population of *T*. *cruzi* ingested with a bloodmeal shows a 90% reduction in the AM within 24 hours [25]. Although parasite reduction is independent of the bacterial population [26], *T*. *cruzi* infection induces the expression of immune factors including antimicrobial peptides (AMPs), nitric oxide, phenoloxidase, lectins, and protease inhibitors [27–29], suggesting a direct interaction or competition for resources [24,30,31].

Infections with trypanosomes and bacteria trigger a tissue specific immune response in *R*. *prolixus;* experimental infection with *T*. *cruzi* and *Trypanosoma rangeli* modulate the expression of AMPs depending on the infection timepoint, tissue (Fat Body (FB) or Midgut (MG)), the trypanosome strain, and the dominant microbiome [21,30–33]. In general, *T*. *rangeli* infections reduce the expression of Lysozyme-B (Lys-B) and Prolixicin, and induce the expression of Defensin-C (Def-C) in the MG; while infections with the *T*. *cruzi* dm28c strain induces Def-C and Prolixicin in the MG [31,32]. Insects fed on blood infected with Gram-positive bacteria showed higher transcription of Def-A, Def-B, Prolixicin, Lys-A in the AM; and of Def-C in the Posterior midgut (PM); and reduced transcription of Prolixicin in the PM 24h after feeding [34]. Feeding with blood containing Gram-negative bacteria induces the transcription of Def-B and Def-C in the AM; of Lys-A in the PM; and reduced transcription of Prolixicin in the PM, and Lys-B in the AM 24h after feeding [34]. Similar immune responses have been reported in *R*. *prolixus* injected intrathoracically with a combination of Gram-positive and Gram-negative bacteria [32,33,35,36]. Within 24h of the infection, Def-A, Def-B, and Lys-A transcripts increase in the MG, while Def-A, Def-B, Prolixicin, and Lys-B are induced in the FB. These results confirm an overall and robust activation and regulation of the innate immune system of *R*. *prolixus*, but the pattern of AMP expression does not follow the traditional separation of Toll and IMD regulated expression.

When the genome of *R*. *prolixus* was published several elements of the IMD pathway were reported as “missing”, and the authors questioned whether this pathway was functional in this insect [37]. Similarly, a reduced IMD pathway was reported in the genomes of other hemimetabolous insects including the pea aphid (*Acyrthosiphon pisum*) [13], the bedbug (*Cimex lectularius*) [38] and the head louse (*Pediculus humanus*) [39]. These researchers proposed that a reduced or inactive IMD pathway was an adaptation to prevent harming the obligate beneficial bacterial symbionts [13,37].

The *R*. *prolixus* genome was reported to lack genes encoding immune deficiency (IMD), Fas-associated Death Domain (FADD), death-related ced-3/Nedd2-like (DREDD), the I-ᴋΒ kinase β (IKKβ), Cylindromatosis (CYLD), Caspar (a negative regulator of the IMD pathway), and the E2 ubiquitination complex (Effete, Uev1A, and Bendless). Despite the apparent absence of these highly conserved members of the IMD pathway, transcriptome studies have reported the expression of transcripts of Relish and a potential Caspar ortholog [40], and gene expression data on AMPs putatively regulated by the IMD pathway are expressed [32,36]. Whether the IMD pathway functions through novel proteins linking existing IMD pathway components, or through a non-canonical pathway, is unknown. Using a combination of bioinformatics and wet bench studies, we have identified most of the “missing” orthologs of the IMD pathway in *R*. *prolixus*. In addition, we have silenced Relish to demonstrate that the IMD pathway in *R*. *prolixus* is similar to that of most holometabolous insects. These data indicate that the IMD pathway is present and inducible in *R*. *prolixus*, and that it is activated preferentially by Gram-negative bacteria.

## Materials and methods

### Ethics statement

All animal care and experimental protocols were conducted following the guidelines of the institutional care and use committee (Committee for Evaluation of Animal Use for Research from the Federal University of Rio de Janeiro), which are based on the National Institutes of Health Guide for the Care and Use of Laboratory Animals (ISBN0-309-05377-3). The protocols were approved by the Committee for Evaluation of Animal Use for Research (CAUAP) from the Federal University of Rio de Janeiro, under registry number 115/13. Technicians dedicated to the animal facility at the Institute of Medical Biochemistry (Federal University of Rio de Janeiro) carried out all aspects related to rabbit husbandry under strict guidelines to ensure careful and consistent handling of the animals.

### Insect rearing

A colony of *R*. *prolixus* is maintained in the insectary of the Institute of Medical Biochemistry at the Federal University of Rio de Janeiro. These insects were fed on live rabbits at three-week intervals and maintained at 28°C and 80–90% relative humidity under a photoperiod of 12h of light and 12h of dark. Recently moulted (1–2 days) fifth instar nymphs were used in the experiments and were maintained under the standard rearing conditions described.

### IMD pathway homolog search

A combination of strategies was used to identify candidate orthologs of “missing” IMD pathway genes in *R*. *prolixus*. We used multiple reciprocal BLAST searches and hidden Markov models (HMM) to create a non-redundant list of candidate orthologs in *R*. *prolixus* [12]. We built an IMD pathway genes database gathering sequences from *Flybase*, III-DB, ORTHO-MCL, Insect-base, I5k, Immuno-DB, and Beebase [41–48].

BLASTn, BLASTp, and tBLASTx searches were performed using amino acid and nucleotide sequences from the IMD pathway genes database as queries against the *R*. *prolixus* protein gene set-C3.1 and against published transcriptomes [40,49]. Matches with significant similarity (e-values < 1x10^-5^, identity > 20%, and query coverage > 50%) were used in reciprocal BLAST searches against a data set comprising 142 arthropod gene-sets using information from Insect-base, IK-5, Flybase, Ensemble genomes, and NCBI transcriptome projects. Candidate orthologs were assessed for conserved protein domain architecture using NCBI Conserved Domain Search and compared with canonical IMD protein domain architecture.

Subsequently, HMM profile searches were performed by building multiple alignments of candidate IMD pathway ortholog proteins from various arthropods. Sequences were selected to avoid taxonomic bias, using crustaceans, arachnids, hemimetabolous and holometabolous insects. The accession numbers for the full sequences can be found in Supplementary File 1 (S1 File) that was used in the phylogenetic analyses. Multiple alignments were built using MUSCLE algorithm and manually edited on MEGA 7.0 software [50,51]. The HMM profiles were built with HMMER 3.1 using default settings and used to search the *R*. *prolixus* protein gene set-C3.1 and protein translated transcriptomes using default settings. HMM profile ortholog sequences were retrieved from KEGG, I5K, Ensemble, and NCBI genome and transcriptome sequencing projects [47,52,53].

### PCR and sequencing of IMD pathway homolog candidates

*Rhodnius prolixus* candidate gene sequences identified using these procedures were retrieved from Vector Base and transcriptome assemblages [40,49,54]. Primers were designed for use in PCR to confirm the transcription of these genes using cDNA templates derived from fat body tissues from immune challenged insects [36]. All reactions used the ABM 2x PCR Taq Mastermix (ABM, Richmond, Canada). Sequences were deposited in GenBank under accession numbers MH484616 to MH484621. Sequences, PCR conditions, and primers used are listed in Supplementary File 1 (S1 File).

### Phylogenetic analysis

Multiple protein alignments with complete sequences or conserved domain sequences were made for “missing” members of the IMD pathway: FADD, DREDD, IAP2, Effete, Bendless, Uev1a, Caspar, CYLD, and IKKβ. Representative arthropod sequences were used if they had been part of other immune studies, had been functionally characterized, or had been manually annotated. Sequences that only had automatic annotations were not used to construct these trees. Human tumour necrosis factor receptor network homologs to IMD pathway genes were used as outgroup sequences. Multiple alignments were made using MUSCLE default parameters in the program MEGA 7.0 [51] (S1 File). Maximum Likelihood analyses were done using IQ-TREE. Best-fit models for each gene alignment were selected based on the Bayesian Information Criterion (BIC). Branch support was assessed by 1000 ultrafast bootstrap replicates and with the Sh-aLTR test.

### dsRNA target selection and synthesis of double-stranded RNA

We performed a selective analysis of rpRelish sequences to reduce the off-target silencing caused during dsRNA injections. The rpRelish cDNA sequence (Genbank sequence KP129556.1) was divided into 19 nucleotide fragments and used as queries with BLASTn against the *R*. *prolixus* transcripts-C3.1 data-set retrieved from Vectorbase. Any hits against genes other than rpRelish larger than 17 nucleotides, > 90% identity, and < 3 gaps were mapped to the rpRelish cDNA and a region with the least number of potential off-target silencing fragments was selected to amplify a 564 bp fragment. For a silencing control, the *Arabidopsis thaliana* AINTEGUMENTA (ANT) gene was subjected to the same analysis and primers were designed to amplify a 516 bp fragment. All primers are listed in S2 Table (S2 Table). PCR used cDNA templates from previous experiments done in our research group and were derived from the fat body of insects challenged with *Escherichia coli* and *Micrococcus luteus* [36] and the products were ethanol precipitated and sequenced. The same primers with an added 5’ incorporated T7 polymerase binding site were used to generate the template required to generate dsRNA. These PCR products were ethanol precipitated and sequenced, and then were used as templates to synthesize double-stranded RNA (dsRNA) as previously described [55]. dsRNA synthesis products were ethanol precipitated, visualized on 1% agarose gel, and quantified on a Nanodrop 1000 spectrophotometer v. 3.7 (Thermo Fisher Scientific).

### RNAi experiments

Intrathoracic injections of dsRNA generated from *R*. *prolixus* Relish, or the ANT gene, were performed on 5^th^ instar insects 1–2 days after moulting. dsRNA was resuspended in 0.9% sterile saline solution and insects were injected with 1 μL containing 2.5 μg of dsRNA using a 10 μL Hamilton syringe.

### Immune challenge

Four days after dsRNA injections, insects were immune challenged by the intrathoracic injection of Gram-negative bacteria (*Enterobacter cloacae*) or Gram-positive bacteria (*Staphylococcus aureus*). Glycerol stocks from these bacteria were grown in Lysogeny Broth (LB) overnight at 200 rpm and 37°C. The liquid cultures were centrifuged, and the pellet was washed 2 times in PBS (137 mM NaCl; 2,7 mM KCl; 10 mM sodium phosphate at pH7.2). The concentration of bacteria was calculated by spectrometry at 600nm on a Shimadzu UV-2550 spectrophotometer. Insects were injected with 2 μL (~10^6^ total bacteria) using a 10 μL Hamilton syringe. Control insects were injected with 2 μL of sterile PBS. Insects were dissected in PBS and fat body tissues were extracted 8 hours after the immune challenge. Each treatment had 5 replicates of 3 pooled fat bodies.

### Tissue isolation, RNA extraction, and cDNA synthesis

Total RNA from dissected FB tissues was extracted using TRizol reagent (Invitrogen, Carlsbad, CA, US) following manufacturer’s recommendations. DNAse treatment was performed with TURBO DNA-free Kit (Ambion). The samples were quantified on a Nanodrop 1000 spectrophotometer v. 3.7 (Thermo Fisher Scientific). First strand cDNA synthesis was performed in 20 μL reactions containing 2.0 μg total RNA using an oligo dT primer (MG) with the OneScript cDNA Synthesis Kit (ABM, Canada) and an extension time of 50 minutes. The subsequent cDNA was diluted 1:50 with DEPC water.

### Quantitative real-time PCR

Quantitative real-time PCR (qPCR) was used to assess rpRelish silencing on the expression of selected AMPs known to have a differential expression in fat body or hemocoel after infection with bacteria or protozoans. Primers were designed, or retrieved from the literature, for: α-Tubulin, rpRelish, rpCaspar, Prolixicin, Lysozyme-B, Defensin-A, and Defensin-C (S3 Table). All reactions contained 4.4 μL of cDNA, 300mM of each primer, and 5 μL of PerfeCTa SYBR Green Super Mix (Quanta Biosciences, Gaithersburg, MD, US) in a final volume of 10 μL. qPCR was performed on a LightCycler96 thermal cycler (Roche diagnostics, Mannheim, Germany). The qPCR conditions used were: 95°C: 3 min, 40 cycles of 95°C: 15 s and 60°C: 30 s, followed by a melt curve analysis to confirm the specificity of the reaction. No-template controls were included with each primer set to verify the absence of exogenous DNA and primer-dimers. Relative differences in transcripts levels were calculated using the 2^– ΔΔ^ Ct method [56–58] with α-Tubulin (RPRC003295) as the reference gene. PCR efficiencies (E) for each primer were determined using the slope of a linear regression mode.

### Statistical analysis

All treatments were tested for normality using the Shapiro-Wilk normality test and compared using the unpaired Student’s T–test or Mann Whitney U Test. Calculations and graphs were made using R 3.0.14 and JMP 13.1. P-values lower than 0.05 were considered as evidence for difference between treatments. Relative transcript levels were expressed as means with whiskers representing ± SEM.

## Results

### *Rhodnius prolixus* IMD pathway orthologs

**Fig 1:**
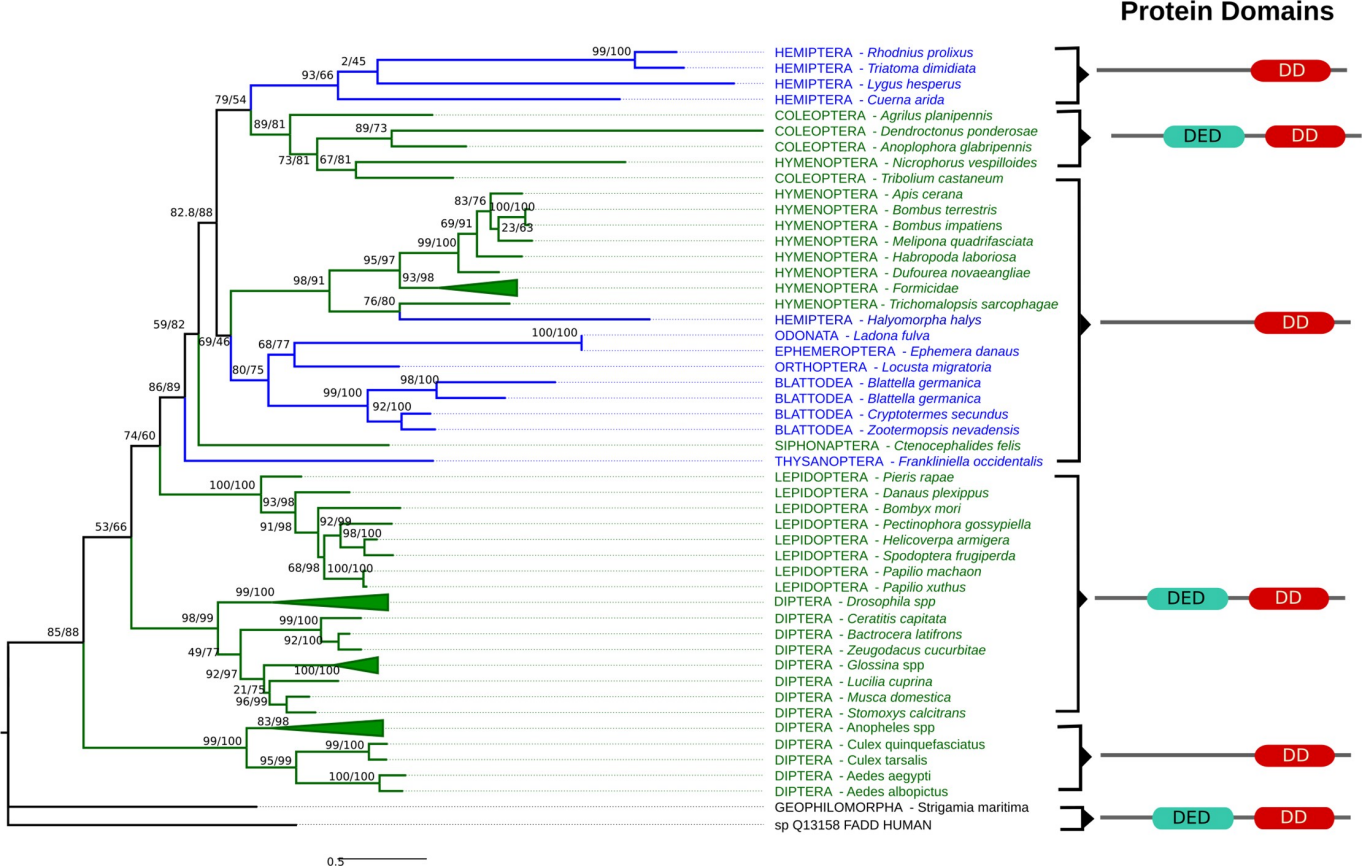
FADD Maximum likelihood trees in selected arthropods. rpFADD forms a common clade with other hemimetabolous insects, coleopterans and hymenopterans. Similar to FADD reported in mosquitoes, rpFADD only has a Death Domain (DD). The Death Effector Domain (DED) present in other orthologs was not detected in rpFADD or mosquitoes. Formicidae, *Drosophila* and *Anopheles* clades are collapsed. The human FADD ortholog was used as the outgroup.

**Fig 2:**
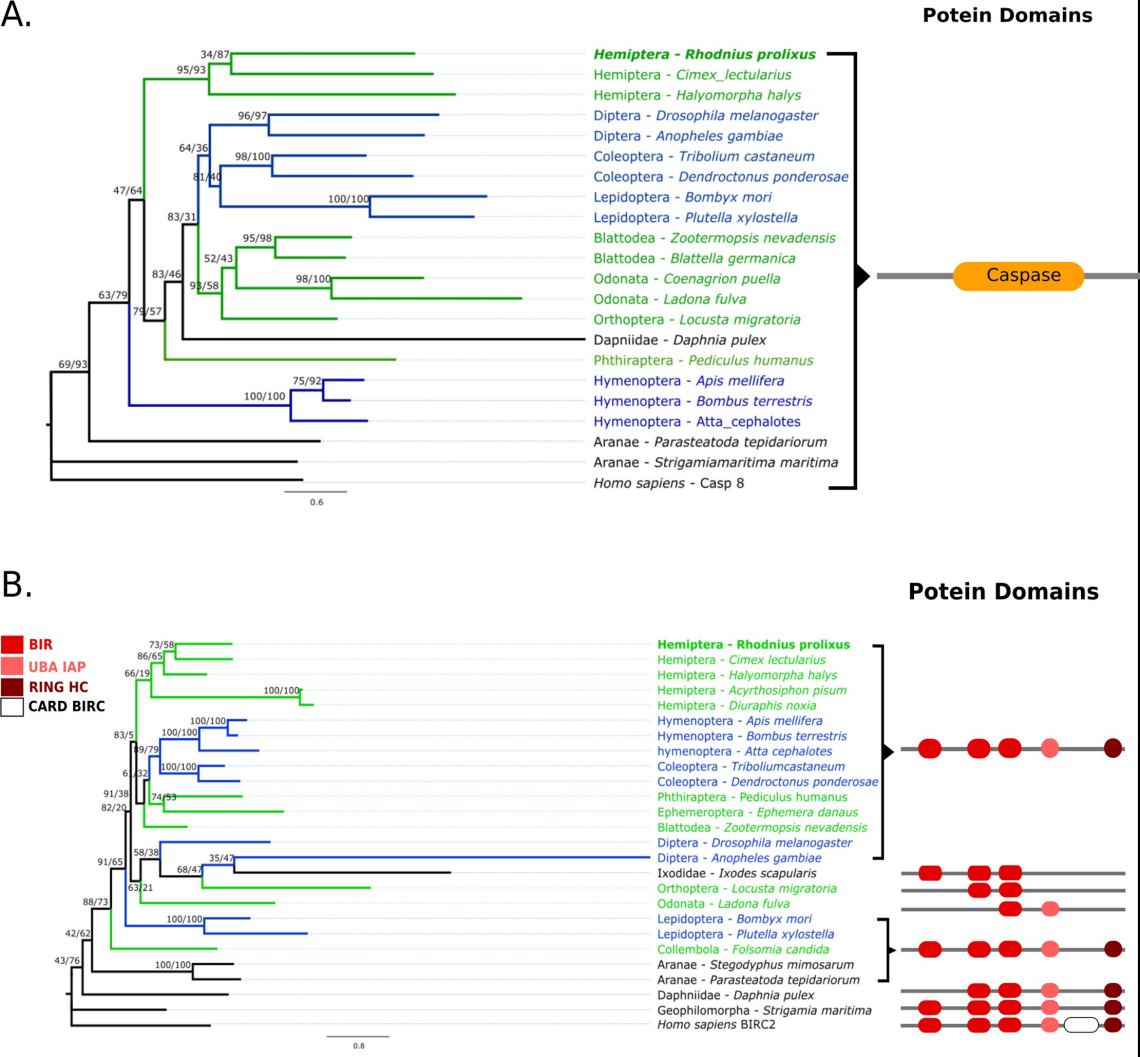
DREDD and CASPAR Maximum likelihood phylogenetic trees in selected arthropods. (A) rpDREDD forms a clade with other hemipterans but is distinct from other hemimetabolous insects (green). (B) rpCASPAR forms a clade with other paraneopteran insects except for aphids which form a distinct clade basal to most insects. The human Caspase 8 and FAF1 were used as outgroups to build the ML trees.

**Fig 3:**
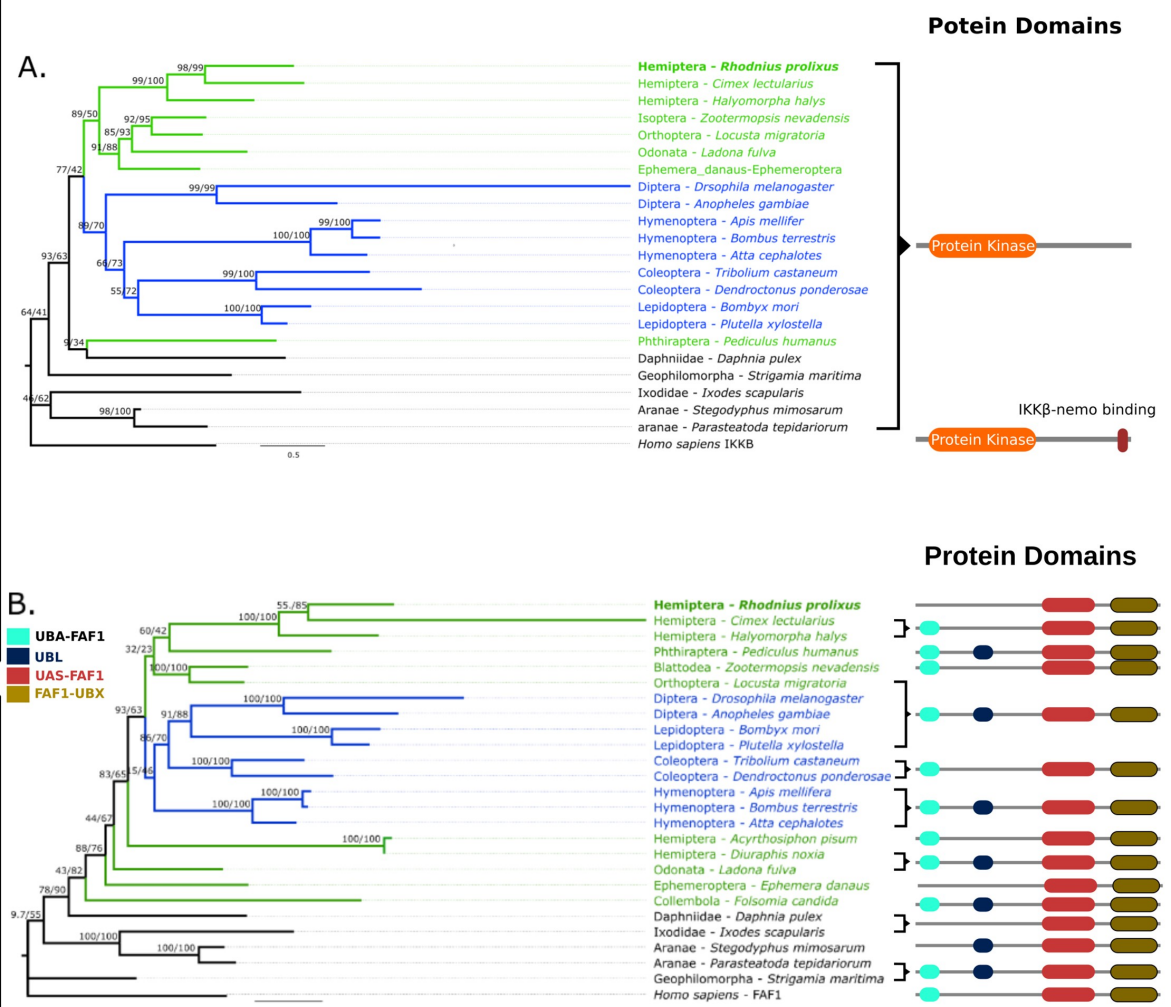
IKKβ and IAP2 Maximum likelihood phylogenetic trees in selected arthropods. (A) rpIKKβ forms a clade with other hemimetabolous insects (except *P*. *humanus)* and is a sister clade to all other insects, but different from other arthropod groups. (B) rpIAP2 forms a clade with other hemipterans and is a sister clade to all hymenopterans and coleopterans insects. The human IKKβ ortholog was used as an outgroup to build the ML trees.

**Fig 4:**
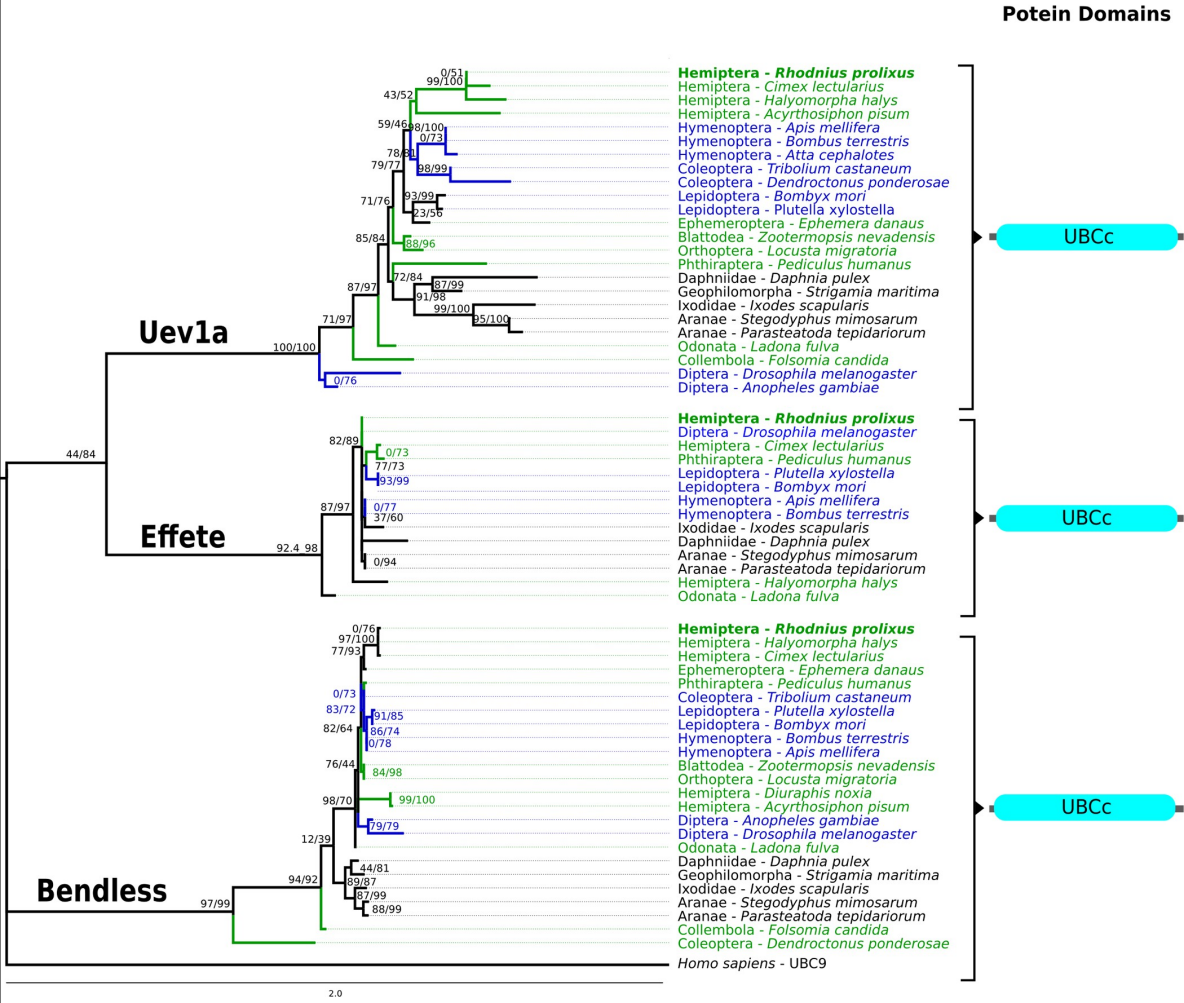
Uev1a, Effete, and Bendless Maximum likelihood phylogenetic trees in selected arthropods. These ubiquitin conjugating enzymes share an UBCc domain that constitutes most of their structure. Orthologs of each of these proteins are contained in a single clade separate from the other protein clades. The human UBC9 was used as outgroup to build the ML trees.

**Fig 5:**
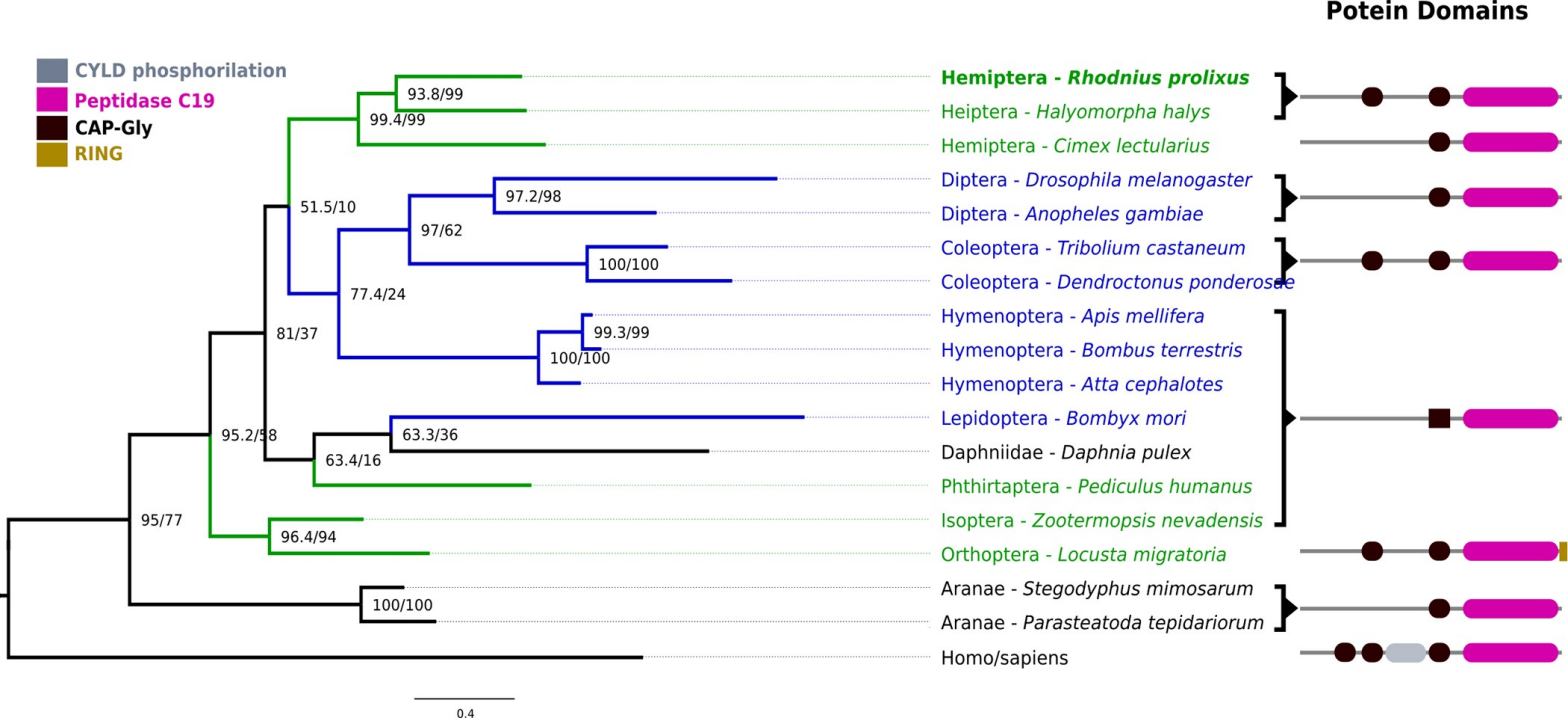
CYLD Maximum likelihood phylogenetic tree in selected arthropods. Hemimetabolous insects are divided among three clades, comprising hemipterans, phthirapterans, and paraneopterans. Most holometabolous insects form a clade that clusters with the hemipteran clade including rpCYLD. The human UBC9 was used as outgroup to build the ML trees.

IMD pathway candidate orthologs were found using reciprocal BLAST and HMM-profile searches. (Table 1). rpFADD (RPRC013858), rpDREDD (GECK01002741.1), rpCaspar (RPRC001459), rpIKKβ (GECK01053880.1), rpIAP2 (RPRC007068), rpEffete (RPRC005317), rpUev1a (RPRC011375), rpBendless (RPRC011790), and rpCYLD (GECK01020525.1) orthologs were found using both search strategies. We did not find candidate orthologs for IMD or Kenny. We confirmed that all orthologs were transcribed using PCR amplification of cDNAs generated from fat body tissues of immune activated insects or by finding their transcripts in published transcriptomes [40,49]. Two orthologs, rpDREDD and rpIKKβ, not currently annotated in the *R*. *prolixus* genome could not be amplified using PCR, but were found in published transcriptomes. rpCYDL is partially annotated in the *R*. *prolixus* genome (RPRC014665) but spans 3 contigs that are assembled in opposing directions, breaking the gene into 3 unlinked fragments. Newly identified putative *R*. *prolixus* orthologs of each gene contain regions that resemble highly conserved architecture and domains of known IMD pathway proteins in other organisms (Figs 1–5) based on sequence alignments (S1 File).

**Table 1:** *Rhodnius prolixus* IMD pathway orthologs, BLASTp search best hit results, and hidden Markov model profile search results.

	BLASTp search results			HMM profile search results
*R*. *prolixus* ortholog and accession numbers	Best-match	Protein name	e-value	e-value
FADD (RPRC013858, GECK01061114.1, MH484617)	*Aethina tumida*	FADD	1.0x10^-8^	1.8x10^-15^
DREDD (GECK01002741.1)	*Oncopeltus fasciatusfasciatus*	DREDD	6.0x10^-40^	1.1x10^-84^
CASPAR (RPRC001459, GECK01051516.1 MH484616)	*Locusta migratoria*	CASPAR	3.0x10^-169^	7.8x10^-201^
IKKβ (GECK01053880.1)	*Cimex lectularius*	IKKβ	3.0x10^-161^	3.8x10^-165^
IAP2 (RPRC007068, GECK01058463.1, MH484621)	*Oncopeltus fasciatus*	IAP2	0.0	1.4x10^-168^
Effete (RPRC005317, GECK01025104.1, MH484619)	*Anoplophora glabripennis*	Effete	3.0x10^-105^	3.1x10^-101^
Uev1a (RPRC011375, GECK01033612.1, MH484620)	*Cimex lectularius*	Uev1a	2.0x10^-98^	1.2x10^-85^
Bendless (RPRC011790, GECK01105599.1, MH484618)	*Halyomorpha halys*	Bendless	1.0x10^-105^	7.6x10^-96^
CYLD (GECK01020525.1)	*Dystrichothorax novaeangliae*	CYLD	0.0	0.0

BLASTp searches used protein sequences against a custom arthropod database. BLASTp Identity percentage and query coverage are over 25% and 73% respectively, for the results shown. HMM protein profile searches were done against a database comprised of the *R*. *prolixus* gene set version 3.1 and selected sequences from transcriptomes. The sequences used for the BLAST searches were from Vector-Base (sequences starting with RPRC), antennal transcriptome [49] (sequences starting with GECK), or sequences generated in this study (Sequences starting with MH). If the sequences were different among sources then the transcriptome sequences were used in the BLAST searches.

To evaluate the evolutionary relationships of *R*. *prolixus* IMD pathway orthologs ML phylogenetic trees with arthropod proteins sequences were built. We included all IMD pathway orthologs found in hemipterans even though no IMD pathway genes have been characterized functionally in this group with the exception of Relish in *R*. *prolixus* [37]. In trees for FADD, DREDD, Caspar, IKKβ, IAP2, and CYLD, non-insect arthropods are basal to insects and holometabolous and hemimetabolous insect orthologs are found in mixed clades (Figs 1–3 and Fig 5). Caspar and IKKβ trees share a similar topology with most hemimetabolous insects as a sister clade to all holometabolous insects (Fig 2B and Fig 3A). Uev1a, Effete, and Bendless orthologs are contained in a single clade separate from the other protein clades. These proteins have almost identical, and extremely conserved, sequences that does not allow a determination of their evolutionary relationships (Fig 4).

### Fas-associated protein with death domain ortholog (FADD)

*Rhodnius prolixus* FADD (rpFADD) has a Death Domain shared with *Drosophila melanogaster* and *Homo sapiens* orthologs. The Death Effector Domain present in most orthologs was not detected in *R*. *prolixus*. However, a similar architecture was reported for FADD found in other hemimetabolous insects, most hymenopterans insects, fleas, and mosquitoes. rpFADD clusters with other hemimetabolous FADD orthologs and are in a common clade with hymenopterans and coleopterans.

### Death-related ced-3/Nedd2-like protein ortholog (DREDD)

*Rhodnius prolixus* DREDD (rpDREDD) has a caspase domain that is conserved in all DREDD orthologs including the human ortholog. ML trees show rpDREDD and hemipteran DREDD orthologs in a clade sister to the majority of insects (Fig 2A). Interestingly, hemipterans and hymenopterans form separate clades from other hemimetabolous and holometabolous insects, respectively.

### FAF1 ortholog (CASPAR)

*Rhodnius prolixus* CASPAR (rpCASPAR) has a UAS family FAS-associated factor 1 domain and a UBQ superfamily domain similar to *D*. *melanogaster* CASPAR. This protein is shorter than other orthologs and lacks a UBA-FAF1 and UBL domain present in other (but not all) orthologs. The ML-tree of this gene shows rpCASPAR in a clade with other hemipteran and paraneopteran insects (Fig 2B). Interestingly, aphid homologs are in a separate clade that is basal to other insects.

### The inhibitor of nuclear factor kappa-β kinase ortholog (IKKβ)

The IKKβ ortholog *in R*. *prolixus* (rpIKKβ) has a catalytic Serine/Threonine kinase, Inhibitor of Nuclear Factor-Kappa B Kinase beta domain present on all IKKβ orthologs. The ML phylogenetic tree for these proteins shows holometabolous and hemimetabolous insects in separate clades (Fig 3A). rpIKKβ is within the latter clade. The human louse *P*. *humanus* is the only hemimetabolous insect that deviates from this pattern, forming a basal clade to these two clades.

### Inhibitor of apoptosis 2 ortholog (IAP2)

The IAP2 ortholog in *R*. *prolixus* (rpIAP2) has the same domain architecture found in *D*. *melanogaster* IAP2 protein. rpIAP2 has 3 BIR domains, 1 UBA domain, and 1 RING-HC domain. The ML tree of this protein shows a clade with all insect IAP2 orthologs (Fig 3B). Hemimetabolous and holometabolous orders form mixed clades that have strong branch support only at the order level. rpIAP2 is in a clade with all other hemipterans.

### Effete, Bendless, and Uev1a orthologs

Effete, Bendless, and Uev1a are ubiquitin conjugating enzymes that share a UBC domain. Reciprocal BLAST searches indicate that RPRC005317, RPRC011375, and RPRC011790 could be either rpEffete, rpUev1a, or rpBendless orthologs. The resulting BLAST hits for the three searches were very similar for different metrics (*e*.*g*. low e-values and high identity). Therefore, a ML tree was built with orthologs of these 3 genes to elucidate their identity (Fig 4). The topology of this tree confirms the HMM-profile searches results, separating the 3 orthologs into different clades (RPRC005317 within rpEffete clade, RPRC011375 within rpUev1a clade, and RPRC011790 within rpBendless clade). Effete and Bendless orthologs are in single clades but the internal branches are poorly supported, likely due to the high protein sequence similarity among these three molecules (Identity percentage >98%).

### Ubiquitin carboxyl-terminal hydrolase Cylindromatosis (CYLD)

The Cylindromatosis CYLD ortholog in *R*. *prolixus* (rpCYLD) has a similar domain architecture to the Drosophila CYLD, with 2 CAP-Gly domains and 1 Peptidase-C19 domain. The ML-tree shows rpCYLD in a clade with other hemipterans, sister to all holometabolous insect CYLD orthologs (Fig 5).

### Differential AMP expression in the fat body

**Fig 6:**
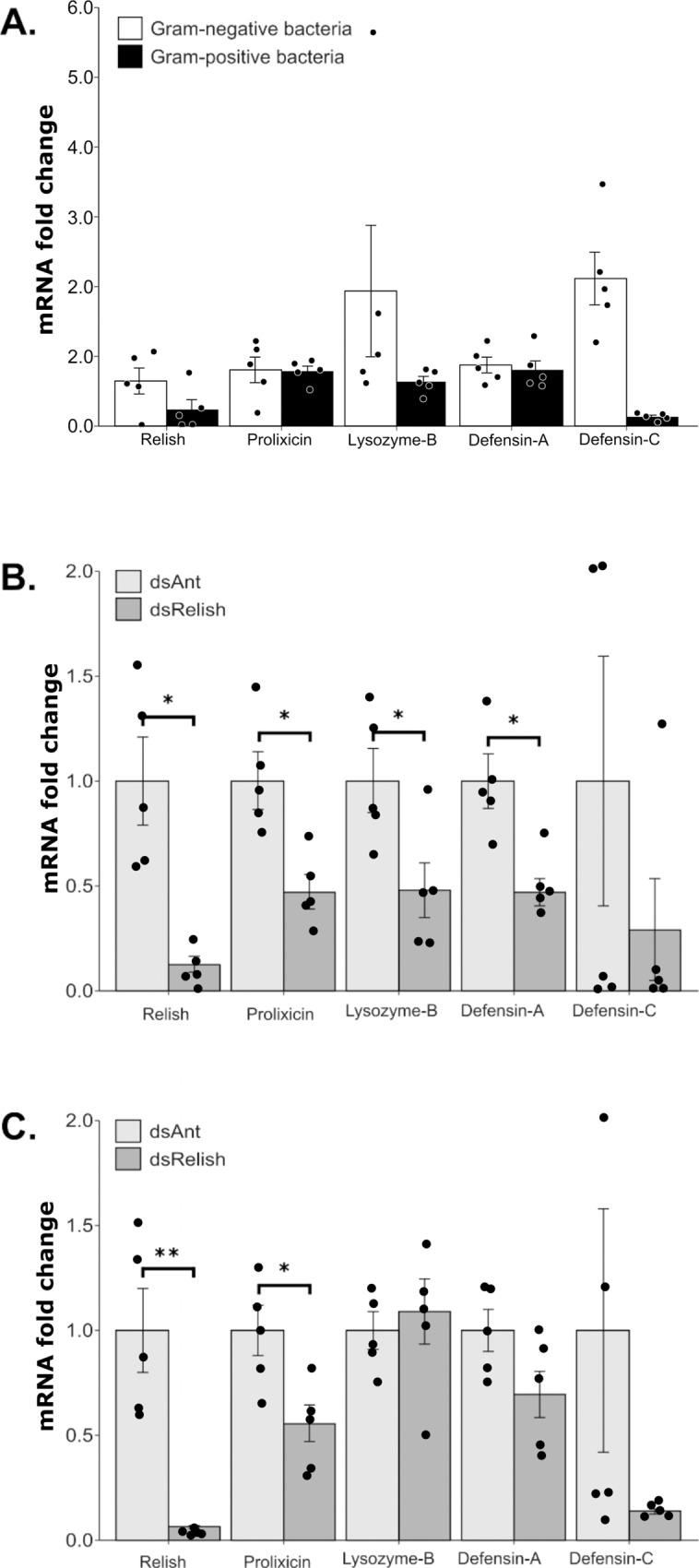
Fat body AMP expression in 5^th^ instar *Rhodnius prolixus* infected with Gram-negative and Gram-positive bacteria. (A) Relative AMP expression in nymphs injected with *Enterobacter cloacae* (Gram-negative) and *Staphylococcus aureus* (Gram-positive) bacteria. Relative expression levels in each gene used levels in the PBS injected insects as the second calibrator, which is assigned a value of 1 in all comparisons as described in [57]. The effect of silencing rpRelish was assessed in insects injected with **(B)** *Enterobacter cloacae* (Gram-negative bacteria) or **(C)** *Staphylococcus aureus* (Gram-positive bacteria). Insects were injected with 2.5 μg of dsRNA complementary to Relish mRNA or ANT, a plant gene that serves as a dsRNA injection control. Four days later insects were injected intrathoracically with 10^6^ bacteria. AMP transcripts were measured 8h after injection with bacteria. Expression analysis used the ΔΔCT method [57] and data are presented as fold differences between dsRelish and dsANT silenced insects. Bars represent the mean transcript levels ± SEM in 5 replicates of 3 pooled fat bodies. Means were compared using the unpaired Student’s T–test. * P < 0.05, ** P < 0.01. Treatments that did not follow a normal distribution (Lys-B in Gram-negative bacteria injection and Def-C in dsANT injection) based on the Shapiro-Wilk normality test (p-values > 0.05, S1 Table), were compared with the Mann Whitney U Test.

The expression of AMPs in the fat body of *R*. *prolixus* was compared 8h after intrathoracic injection of Gram-negative or Gram-positive bacteria. Transcript levels for each AMP were compared with the levels expressed in PBS injected insects serving as the second calibrator (ΔΔCT [57,58]). The expression of all AMPs was similar after injection with Gram-positive or Gram-negative bacteria (Fig 6A). Only Defensin-C showed a significant induction after injection with Gram-negative bacteria (CI_95_ 1.01–3.16) and a suppression after injection of Gram-positive bacteria (CI_95_ 0.06–0.19). rpRelish levels were lower in insects injected with Gram-positive bacteria compared with insects injected with PBS (CI95−0.12–0.62)

### The effect of silencing rpRelish on AMP transcription

AMP transcript levels were measured 8h after intrathoracic inoculation of bacteria into insects that had received dsRNA. In these experiments, AMP expression levels were compared between insects that had been injected with dsANT or dsRelish. rpRelish knockdown reduced rpRelish expression by 88% compared with dsANT injected insects that later were injected with Gram-negative bacteria, and by 94% compared with dsANT injected that later were injected with Gram-positive bacteria (Fig 6B and 6C).

In rpRelish silenced insects injected with Gram-negative bacteria, Prolixicin, Lysozyme-B, and Defensin-A expression decreased to 47% (CI_95_ 24% - 70%), 48% (CI_95_ 11% - 84%) and 46% (CI_95_ 28% - 66%) respectively of their dsANT injected controls. Defensin-C transcripts levels showed a high degree of variation, with no evidence of differential transcription between dsANT and dsRelish treated insects (Fig 6B). After infection with Gram-positive bacteria, only Prolixicin showed significant changes in expression with transcript levels reduced to 55% (CI_95_ 31%-80%) of dsANT injected insects (Fig 6C).

## Discussion

### Homolog search

Using a combination of reciprocal BLAST searches and the construction of taxonomically unbiased HMMs we found novel orthologs that represent most of the “missing” members of the IMD pathway in *R*. *prolixus*. Hidden Markov model searches outperformed reciprocal BLAST searches, but both strategies found the same candidate genes if they were annotated in the genome. Zumaya-Estrada *et al* [59] used similar homology search strategies and found several IMD pathway genes [59], while Ribeiro *et al* found a Caspar homolog in a midgut transcriptome [40]. In our analysis of published transcriptomes, we found 3 additional genes that are not currently annotated in the *R*. *prolixus* genome. We were unable, however, to find orthologs for IMD and Kenny. These data suggest that the annotated gene list from *R*. *prolixus* is incomplete. Software regularly used to annotate genomes uses *ab-initio* approaches based on the structure of known genes. Hemipterans seem to have an unusual gene-structure with genes made of shorter exons than other insects [60]. Consequently, *ab-initio* approaches based on non-hemipteran insects might not provide accurate gene models. Gene prediction software such as Augustus and MAKER can be trained to generate relevant gene models for hemipterans as they allow approaches to combine data from transcriptomes, EST, and genomes from taxonomically closer organisms [61,62]. Therefore, the assumed absence of IMD pathway genes in many arthropods could be explained either by the absence of orthologs or by the difficulty in finding these genes. In this study, we adapted a strategy from Palmer and Jiggins [12] that combines genomic and transcriptomic data and the use of HMM to better predict distant homologs. It is important to recall, however, that genomic and transcriptomic data are not infallible and that even accurate gene models can fail to predict genes. In the milk weed bug genome project, for example, an IMD gene was only found by classic cloning and the use of degenerate primers after the failure of genomic and transcriptomic sequencing to identify strong candidate molecules.

### Evolution of IMD pathway genes

**Fig 7:**
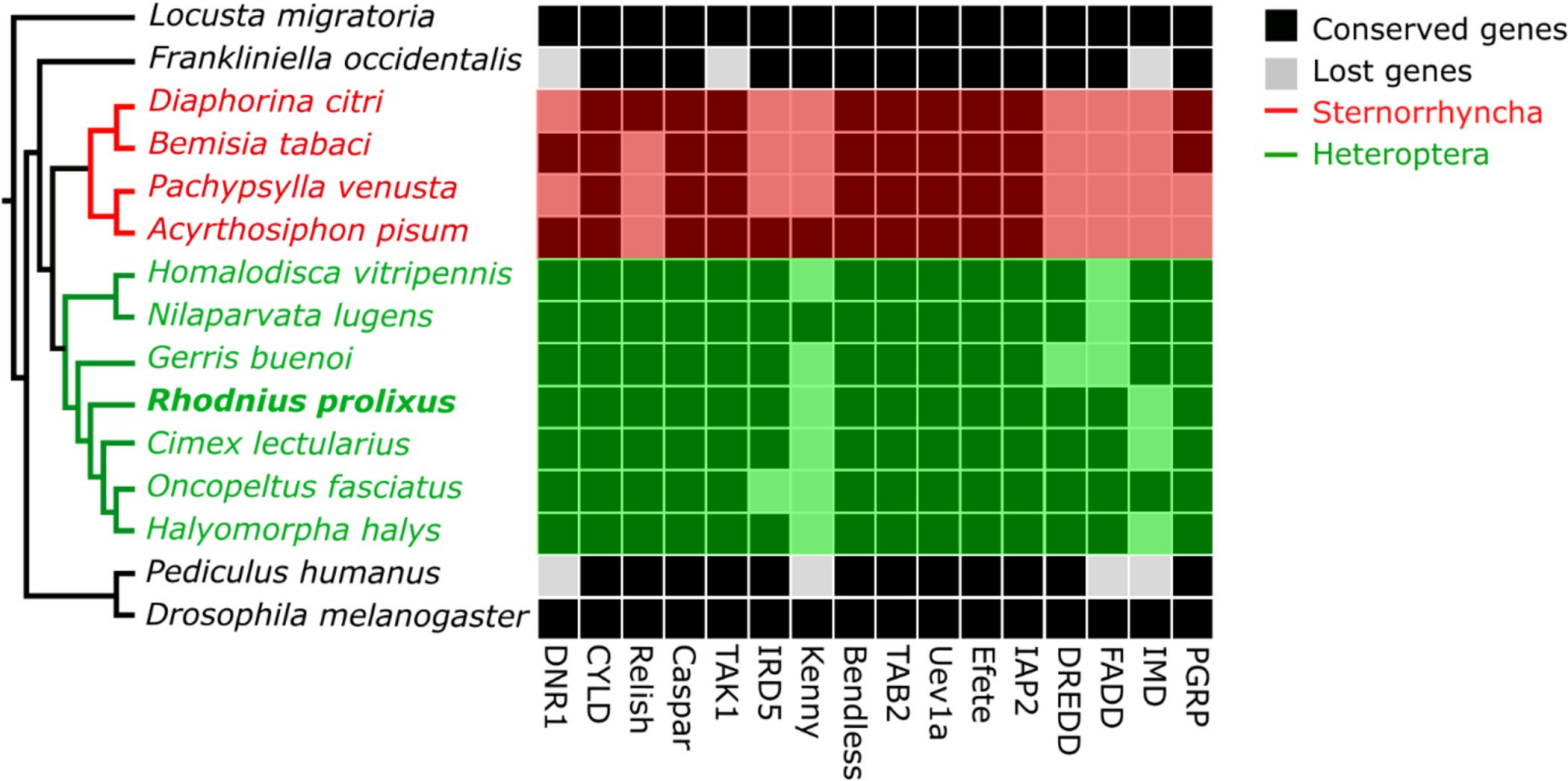
IMD pathway gene orthologs in selected insects. The presence of gene orthologs in several selected holo- and hemimetabolous insects is compared with their phylogeny. Orthologs that are present are shown as black boxes and absent orthologs are shown as white boxes. The tree indicates heteropterans, including *Rhodnius prolixus* in green, the sternorrynchans, including aphids, whiteflies, and scale insects, are in red and holometabolous insects and paraneopeterans are in black. The phylogenetic relationships among the species shown were modified after [70].

The IMD pathway is the most variable immune pathway in arthropods [12,63], and hemipterans are the principal insect group with a reported gene reduction [13,37,59,60,64–67]. After a closer inspection of the IMD pathway in hemipterans, we found that insects in the Sternorrhyncha superfamily (Planthoppers, white flies, and aphids) indeed have a loss of important membrane-proximal signaling genes (PGRP, IMD, FADD, and DREDD, and Relish) and the NF-κβ transcription factor Relish (Fig 7). The absence of these genes could be an adaptation in sternorrhynchans related to the specialized feeding habits and highly specific relationships they have with symbiotic bacteria that provide essential amino acids and nutrients [68,69]. Sternorrhynchans, in general, are missing multiple elements of the IMD pathway, including *D*. *citrii*, which is considered in most phylogenetic trees to be basal to the other members of this group, and *A*. *pisium*, considered to be more derived. On the other hand, we found a very complete pathway for most heteropteran insects (true bugs) and the large milk weed bug, *Oncopeltus fasciatus*, seems to have a complete IMD pathway with orthologs for IMD, FADD, and DREDD. All other heteropterans lack at least one of these genes, and all but the brown planthopper, *Nilaparvata lugens*, are lacking orthologs for Kenny. The absence of IMD itself is especially intriguing since this molecule is central to the operation of the pathway. In Drosophila, IMD interacts with PGRPs and FADD; it is cleaved by DREDD, and K63-ubiquitinated by IAP2 [4]. An alternative activation of the IMD pathway may exist in *R*. *prolixus*. One possibility is that FADD can transduce the IMD pathway signaling in the absence of IMD. Another alternative is the existence of a novel protein that functions as IMD, or the cryptic functioning of another protein acting as IMD. While our methodology to find distant orthologs is very sensitive, we cannot discard the possibility that the absence of IMD is due to further sequence divergence of this gene. The use of biochemical assays will help elucidate these scenarios.

The IMD pathway evolved early in arthropods and orthologs for this pathway are found in crustaceans, polyneopterans, and holometabolous insects. It has been proposed, across a broad range of taxa, that immune related genes are among the most rapidly evolving genes [71,72]. IMD pathway genes show significantly more sequence divergence among insect species than genes in other immune pathways, which might suggest that hemipterans faced different selective pressures in the evolution of this pathway [4,10,12,73]. Indeed, the IMD pathway has stronger positive selection than other pathways in *Drosophila* [74–76] and termites [77]. Genes with evidence of positive selection in these insects are involved in signal transduction (e.g. Relish, Dredd, IKK-β, and DNR1) and it has been hypothesized that pathogens are driving the adaptative evolution of these molecules [77,78]. Hemipterans have a strong association with endosymbionts [79–81] that could trigger higher rates of evolution throughout the whole pathway, but this needs to be addressed formally. One explanation of why the IMD pathway is more variable than other immune pathways and might be more adaptable to symbiotic and pathogenic responses, is that it is not closely linked to developmental processes as is the case of the Toll pathway.

Many of the organisms that have a modified IMD pathway also have specialized diets such as sap feeding in aphids or obligate hematophagy in bedbugs and kissing bugs. It has been proposed that such specialized diets contribute to the evolution of a reduced immune system because i) these diets contain few microorganisms and therefore do not require a robust immune activation, and ii) symbiotic organisms are tolerated by reduced immune systems because they provide nutrients that are absent in the limited diet [82]. Our findings do not fully support these hypotheses in *R*. *prolixus*. Our updated IMD pathway in *R*. *prolixus* resembles those from non-hematophagous insects and is activated during bacterial infections. Furthermore, kissing bugs are gregarious insects that live in non-sterile environments. Feeding is not restricted to sterile blood; early instar nymphs acquire obligate symbionts through coprophagy and hemolymphagy and phytophagy occur in triatomines, challenging the convention that they are strictly hematophagous [83–85]. The evolution of tolerance mechanisms towards bacterial symbionts exist in other insects and do not involve the depletion of immune related genes. Intracellular bacteria in tsetse flies are found in a bacteriosome that downregulates the IMD pathway by producing PGRP-LB [86]. This PGN amidase degrades the PAMP that activates the pathway, therefore protecting the endosymbiont. This mechanism also operates in the weevil *Sitophilus zeamais* [87]. The principal endosymbiont of *R*. *prolixus*, *Rhodococcus rhodnii*, provides B-complex vitamins, similar to the role played by *Wigglesworthia* sp in tsetse flies. *Rhodococcus* sp., however, is extracellular, does not form bacteriosomes, and therefore would require different tolerance mechanisms. It is likely that such tolerance mechanisms would be mediated by the IMD and Toll pathways since silencing of dorsal (Toll pathway transcription factor) increases the bacterial population in the midgut while silencing of Relish induces a reduction in bacterial populations [37].

### IMD pathway control of AMPs

AMPs are regulated directly by the Toll and IMD pathways in arthropods. In *Drosophila*, the Toll pathway is reported to respond to infections with fungi and Gram-positive bacteria while the IMD pathway is activated by Gram-negative bacteria, and each pathway induces the expression of a subset of AMPs. Recent studies, however, indicate that AMP expression can be coregulated by the IMD, Toll, and JAK-STAT pathways [88–90]. In *R*. *prolixus*, we demonstrated that the IMD pathway principally regulates AMP expression against Gram-negative bacteria (Fig 6A and 6B) compared with infections with Gram-positive bacteria (Fig 6A and 6C). The expression of all the AMPs we measured was reduced in insects in which rpRelish expression was reduced using RNAi and posteriorly injected with Gram-negative bacteria, suggesting that the IMD pathway is regulating the levels of all AMPs. Interestingly silencing of rpRelish also regulates AMP expression after infection with Gram-positive bacteria. These results differ from the *Drosophila* model and suggest an interaction between recognition or signaling events of the IMD and Toll pathways. Nishide *et al* reported a strong crosstalk between these pathways in the heteropteran *Plautia stali* [9]. In their model, insects injected with Gram-positive or Gram-negative bacteria upregulated the same AMPs. Furthermore, silencing the Toll or IMD pathways reduced a subset of these AMPs. Maximum suppression of all AMP expression was achieved by co-silencing both pathways. This study and ours using RNAi support the hypothesis of a coregulation of AMP expression by both pathways, but also indicate that one pathway predominately regulates each AMP. Although we did not measure the influence of the Toll pathway in *R*. *prolixus*, the expression patterns of Prolixicin and Defensin-C suggests they are likely regulated by the Toll pathway.

This coregulation concept is not limited to hemimetabolous insects. The expression of Gambicin in *Aedes aegypti* is regulated by transcription factors of the IMD and JAK-STAT pathways [88]. This might be the case with Defensin-C in *R*. *prolixus* as there is a STAT binding site 174bp upstream of the coding region. Defensin-A and Lysozyme-B, however, have a NF-κβ binding site at this same location that could make the regulation of these genes more responsive to Relish or Dorsal than STAT [33]. Surprisingly, Prolixicin expression is dependent on Relish despite not having an obvious NF-κβ binding domains in its promoter region [32] but the discordant expression of AMPs in the fat body and in different sections of the midgut cannot be explained solely by IMD NF-κβ sites. STAT and Toll NF-κβ binding sites may provide additional regulation that accounts for these differences. A systematic analysis of AMP promoters might help determine factors in the co-regulation of AMPs by multiple regulatory pathways.

Regardless of the combination of factors that contribute to the expression and regulation of multiple AMPs, we have identified molecules that fill in many of the reported gaps in the *R*. *prolixus* IMD pathway. In contrast to the conclusions of the *R*. *prolixus* genome paper [36], our data indicate that this pathway is functional and inducible in *R*. *prolixus* and now can be considered very similar to the described IMD pathways of holometabolous insects.

## Supporting information

S1 File Amino acid sequence alignments used to generate the phylogenetic trees and the HMM profiles. Accession numbers are reported alongside the sequences.
(TXT)

S1 Supporting Data mRNA fold change data for AMPs used to build Fig 6. (XLSX)

S1 Table *Rhodnius prolixus* IMD pathway homologs with genes reported in GenBank and Vector Base amplified with the described primers using *Rhodnius prolixus* fat body cDNA. The resulting sequences are available in GenBank under accession numbers beginning with MH.
(DOCX)

S2 Table Primers for the generation of dsRNA for use in silencing assays. Each gene was first PCR amplified with primers in bold. These PCR amplicons then were used as template for a second PCR with primers that included the 5’ T7 promoter (complete sequence). PCR amplification profiles and purification procedures are described in the Methods section.
(DOCX)

S3 Table Primers for quantitative real time PCR. Primers were designed using the PRIMER3, Beacon Designer^TM^, and mFOLD.
(DOCX)

## References

[1] Kimbrell DA, Beutler B. The evolution and genetics of innate immunity. Nat Rev Genet. 2001;2: 256–267. 10.1038/35066006 11283698

[2] Valanne S, Wang J-H, Rämet M. The *Drosophila* Toll signaling pathway. J Immunol. 2011;186: 649–656. 10.4049/jimmunol.1002302 21209287

[3] Myllymäki H, Valanne S, Rämet M. The *Drosophila* Imd signaling pathway. J Immunol. 2014;192: 3455–3462. 10.4049/jimmunol.1303309 24706930

[4] Kleino A, Silverman N. The *Drosophila* IMD pathway in the activation of the humoral immune response. Dev Comp Immunol. 2014;42: 25–35. 10.1016/j.dci.2013.05.014 23721820 PMC3808521

[5] Morin-Poulard I, Vincent A, Crozatier M. The *Drosophila* JAK-STAT pathway in blood cell formation and immunity. JAKSTAT. 2013;2: e25700 10.4161/jkst.25700 24069567 PMC3772119

[6] Lindsay SA, Wasserman SA. Conventional and non-conventional *Drosophila* Toll signaling. Dev Comp Immunol. 2014;42: 16–24. 10.1016/j.dci.2013.04.011 23632253 PMC3787077

[7] Ganesan S, Aggarwal K, Paquette N, Silverman N. NF-κB/Rel proteins and the humoral immune responses of *Drosophila melanogaster*. Curr Top Microbiol Immunol. 2011;349: 25–60. 10.1007/82_2010_107 20852987 PMC3083852

[8] Tanji T, Hu X, Weber ANR, Ip YT. Toll and IMD pathways synergistically activate an innate immune response in *Drosophila melanogaster*. Mol Cell Biol. 2007;27: 4578–4588. 10.1128/MCB.01814-06 17438142 PMC1900069

[9] Nishide Y, Kageyama D, Yokoi K, Jouraku A, Tanaka H, Futahashi R, et al Functional crosstalk across IMD and Toll pathways: insight into the evolution of incomplete immune cascades. Proceedings of the Royal Society B: Biological Sciences. 2019;286: 20182207 10.1098/rspb.2018.2207 PMC6408883 30963836

[10] Lai AG, Aboobaker AA. Comparative genomic analysis of innate immunity reveals novel and conserved components in crustacean food crop species. BMC Genomics. 2017;18: 389 10.1186/s12864-017-3769-4 28521727 PMC5437397

[11] Shaw DK, Wang X, Brown LJ, Chávez ASO, Reif KE, Smith AA, et al Infection-derived lipids elicit an immune deficiency circuit in arthropods. Nat Commun. 2017;8: 14401 10.1038/ncomms14401 28195158 PMC5316886

[12] Palmer WJ, Jiggins FM. Comparative genomics reveals the origins and diversity of arthropod immune systems. Mol Biol Evol. 2015;32: 2111–2129. 10.1093/molbev/msv093 25908671 PMC4833078

[13] Elsik CG. The pea aphid genome sequence brings theories of insect defense into question. Genome Biol. 2010;11: 106 10.1186/gb-2010-11-2-106 20236492 PMC2872869

[14] Kim JH, Min JS, Kang JS, Kwon DH, Yoon KS, Strycharz J, et al Comparison of the humoral and cellular immune responses between body and head lice following bacterial challenge. Insect Biochem Mol Biol. 2011;41: 332–339. 10.1016/j.ibmb.2011.01.011 21296152

[15] Armitage SAO, Peuss R, Kurtz J. Dscam and pancrustacean immune memory—a review of the evidence. Dev Comp Immunol. 2015;48: 315–323. 10.1016/j.dci.2014.03.004 24657209

[16] Chagas C. Nova tripanozomiaze humana: estudos sobre a morfolojia e o ciclo evolutivo do Schizotrypanum cruzi n. gen., n. sp., ajente etiolojico de nova entidade morbida do homem. Mem Inst Oswaldo Cruz. 1909;1: 159–218. 10.1590/S0074-02761909000200008

[17] Snodgrass RE. The principles of insect physiology. Science. 1939;90: 159–159. 10.1126/science.90.2329.159-a 17829328

[18] TEJERA Gr. E. La Trypanosome américaine ou maladie de Chagas au Venezuela. Bulletin de la Société de Pathologie Exotique. 1919;12: 509–513. Available: https://www.cabdirect.org/cabdirect/abstract/19202900678

[19] Chagas disease in Latin America: an epidemiological update based on 2010 estimates. Wkly Epidemiol Rec. 2015;90: 33–43. 25671846

[20] World Health Organization, Savioli L, Daumerie D, World Health Organization Department of Control of Neglected Tropical Diseases. Sustaining the drive to overcome the global impact of neglected tropical diseases: Second WHO report on neglected tropical diseases. Geneva, Switzerland: World Health Organization;

[21] Mello CB, Garcia ES, Ratcliffe NA, Azambuja P. *Trypanosoma cruzi* and *Trypanosoma rangeli*: interplay with hemolymph components of *Rhodnius prolixus*. J Invertebr Pathol. 1995;65: 261–268. 10.1006/jipa.1995.1040 7745280

[22] Whitten MM, Mello CB, Gomes SA, Nigam Y, Azambuja P, Garcia ES, et al Role of superoxide and reactive nitrogen intermediates in *Rhodnius prolixus* (Reduviidae)/*Trypanosoma rangeli* interactions. Exp Parasitol. 2001;98: 44–57. 10.1006/expr.2001.4615 11426951

[23] Azambuja P, Feder D, Garcia ES. Isolation of Serratia marcescens in the midgut of Rhodnius prolixus: impact on the establishment of the parasite Trypanosoma cruzi in the vector. Exp Parasitol. 2004;107: 89–96. 10.1016/j.exppara.2004.04.007 15208042

[24] Castro DP, Moraes CS, Gonzalez MS, Ratcliffe NA, Azambuja P, Garcia ES. *Trypanosoma cruzi* immune response modulation decreases microbiota in *Rhodnius prolixus* gut and is crucial for parasite survival and development. PLoS One. 2012;7: e36591 10.1371/journal.pone.0036591 22574189 PMC3344921

[25] Ferreira RC, Kessler RL, Lorenzo MG, Paim RMM, Ferreira LDL, Probst CM, et al Colonization of *Rhodnius prolixus* gut by *Trypanosoma cruzi* involves an extensive parasite killing. Parasitology. 2016;143: 434–443. 10.1017/S0031182015001857 26818093

[26] Dias F de A, Guerra B, Vieira LR, Perdomo HD, Gandara ACP, Amaral RJV do, et al Monitoring of the Parasite Load in the Digestive Tract of *Rhodnius prolixus* by Combined qPCR Analysis and Imaging Techniques Provides New Insights into the Trypanosome Life Cycle. PLoS Negl Trop Dis. 2015;9: e0004186 10.1371/journal.pntd.0004186 26496442 PMC4619730

[27] Garcia ES, Genta FA, de Azambuja P, Schaub GA. Interactions between intestinal compounds of triatomines and *Trypanosoma cruzi*. Trends Parasitol. 2010;26: 499–505. 10.1016/j.pt.2010.07.003 20801082

[28] Flores-Villegas AL, Salazar-Schettino PM, Córdoba-Aguilar A, Gutiérrez-Cabrera AE, Rojas-Wastavino GE, Bucio-Torres MI, et al Immune defence mechanisms of triatomines against bacteria, viruses, fungi and parasites. Bull Entomol Res. 2015;105: 523–532. 10.1017/S0007485315000504 26082354

[29] Guarneri AA, Lorenzo MG. Triatomine physiology in the context of trypanosome infection. J Insect Physiol. 2017;97: 66–76. 10.1016/j.jinsphys.2016.07.005 27401496

[30] Vieira CS, Mattos DP, Waniek PJ, Santangelo JM, Figueiredo MB, Gumiel M, et al *Rhodnius prolixus* interaction with *Trypanosoma rangeli*: modulation of the immune system and microbiota population. Parasit Vectors. 2015;8: 135 10.1186/s13071-015-0736-2 25888720 PMC4350287

[31] Vieira CS, Waniek PJ, Castro DP, Mattos DP, Moreira OC, Azambuja P. Impact of *Trypanosoma cruzi* on antimicrobial peptide gene expression and activity in the fat body and midgut of *Rhodnius prolixus*. Parasit Vectors. 2016;9: 119 10.1186/s13071-016-1398-4 26931761 PMC4774030

[32] Ursic-Bedoya R, Buchhop J, Joy JB, Durvasula R, Lowenberger C. Prolixicin: a novel antimicrobial peptide isolated from *Rhodnius prolixus* with differential activity against bacteria and *Trypanosoma cruzi*. Insect Mol Biol. 2011;20: 775–786. 10.1111/j.1365-2583.2011.01107.x 21906194

[33] Ursic-Bedoya RJ, Nazzari H, Cooper D, Triana O, Wolff M, Lowenberger C. Identification and characterization of two novel lysozymes from *Rhodnius prolixus*, a vector of Chagas disease. J Insect Physiol. 2008;54: 593–603. 10.1016/j.jinsphys.2007.12.009 18258253

[34] Vieira CS, Waniek PJ, Mattos DP, Castro DP, Mello CB, Ratcliffe NA, et al Humoral responses in *Rhodnius prolixus*: bacterial feeding induces differential patterns of antibacterial activity and enhances mRNA levels of antimicrobial peptides in the midgut. Parasit Vectors. 2014;7: 232 10.1186/1756-3305-7-232 24885969 PMC4032158

[35] Lopez L, Morales G, Ursic R, Wolff M, Lowenberger C. Isolation and characterization of a novel insect defensin from *Rhodnius prolixus*, a vector of Chagas disease. Insect Biochem Mol Biol. 2003;33: 439–447. 10.1016/S0965-1748(03)00008-0 12650692

[36] Ursic-Bedoya RJ, Lowenberger CA. *Rhodnius prolixus*: identification of immune-related genes up-regulated in response to pathogens and parasites using suppressive subtractive hybridization. Dev Comp Immunol. 2007;31: 109–120. 10.1016/j.dci.2006.05.008 16824597

[37] Mesquita RD, Vionette-Amaral RJ, Lowenberger C, Rivera-Pomar R, Monteiro FA, Minx P, et al Genome of *Rhodnius prolixus*, an insect vector of Chagas disease, reveals unique adaptations to hematophagy and parasite infection. Proc Natl Acad Sci USA. 2015;112: 14936–14941. 10.1073/pnas.1506226112 26627243 PMC4672799

[38] Benoit JB, Adelman ZN, Reinhardt K, Dolan A, Poelchau M, Jennings EC, et al Unique features of a global human ectoparasite identified through sequencing of the bed bug genome. Nat Commun. 2016;7: 10165 10.1038/ncomms10165 26836814 PMC4740739

[39] Kirkness EF, Haas BJ, Sun W, Braig HR, Perotti MA, Clark JM, et al Genome sequences of the human body louse and its primary endosymbiont provide insights into the permanent parasitic lifestyle. Proc Natl Acad Sci USA. 2010;107: 12168–12173. 10.1073/pnas.1003379107 20566863 PMC2901460

[40] Ribeiro JMC, Genta FA, Sorgine MHF, Logullo R, Mesquita RD, Paiva-Silva GO, et al An insight into the transcriptome of the digestive tract of the bloodsucking bug, *Rhodnius prolixus*. PLoS Negl Trop Dis. 2014;8: e2594 10.1371/journal.pntd.0002594 24416461 PMC3886914

[41] Bao Y-Y, Qu L-Y, Zhao D, Chen L-B, Jin H-Y, Xu L-M, et al The genome- and transcriptome-wide analysis of innate immunity in the brown planthopper, *Nilaparvata lugens*. BMC Genomics. 2013;14: 160 10.1186/1471-2164-14-160 23497397 PMC3616906

[42] Gramates LS, Marygold SJ, Santos GD, Urbano J-M, Antonazzo G, Matthews BB, et al FlyBase at 25: looking to the future. Nucleic Acids Res. 2017;45: D663–D671. 10.1093/nar/gkw1016 27799470 PMC5210523

[43] Brucker RM, Funkhouser LJ, Setia S, Pauly R, Bordenstein SR. Insect Innate Immunity Database (IIID): an annotation tool for identifying immune genes in insect genomes. PLoS One. 2012;7: e45125 10.1371/journal.pone.0045125 22984621 PMC3440344

[44] Li L, Stoeckert CJ, Roos DS. OrthoMCL: identification of ortholog groups for eukaryotic genomes. Genome Res. 2003;13: 2178–2189. 10.1101/gr.1224503 12952885 PMC403725

[45] Waterhouse RM, Kriventseva EV, Meister S, Xi Z, Alvarez KS, Bartholomay LC, et al Evolutionary dynamics of immune-related genes and pathways in disease-vector mosquitoes. Science. 2007;316: 1738–1743. 10.1126/science.1139862 17588928 PMC2042107

[46] Yin C, Shen G, Guo D, Wang S, Ma X, Xiao H, et al InsectBase: a resource for insect genomes and transcriptomes. Nucleic Acids Res. 2016;44: D801–7. 10.1093/nar/gkv1204 26578584 PMC4702856

[47] Poelchau M, Childers C, Moore G, Tsavatapalli V, Evans J, Lee C-Y, et al The i5k Workspace@NAL—enabling genomic data access, visualization and curation of arthropod genomes. Nucleic Acids Res. 2015;43: D714–9. 10.1093/nar/gku983 25332403 PMC4384035

[48] Elsik CG, Tayal A, Diesh CM, Unni DR, Emery ML, Nguyen HN, et al Hymenoptera Genome Database: integrating genome annotations in HymenopteraMine. Nucleic Acids Res. 2016;44: D793–800. 10.1093/nar/gkv1208 26578564 PMC4702858

[49] Latorre-Estivalis JM, Robertson HM, Walden KKO, Ruiz J, Gonçalves LO, Guarneri AA, et al The molecular sensory machinery of a Chagas disease vector: expression changes through imaginal moult and sexually dimorphic features. Sci Rep. 2017;7: 40049 10.1038/srep40049 28059141 PMC5216343

[50] Kumar S, Stecher G, Tamura K. MEGA7: molecular evolutionary genetics analysis version 7.0 for bigger datasets. Mol Biol Evol. 2016;33: 1870–1874. 10.1093/molbev/msw054 27004904 PMC8210823

[51] Edgar RC. MUSCLE: multiple sequence alignment with high accuracy and high throughput. Nucleic Acids Res. 2004;32: 1792–1797. 10.1093/nar/gkh340 15034147 PMC390337

[52] Kanehisa M, Goto S. KEGG: kyoto encyclopedia of genes and genomes. Nucleic Acids Res. 2000;28: 27–30. 10592173 10.1093/nar/28.1.27 PMC102409

[53] Kersey PJ, Allen JE, Allot A, Barba M, Boddu S, Bolt BJ, et al Ensembl Genomes 2018: an integrated omics infrastructure for non-vertebrate species. Nucleic Acids Res. 2018;46: D802–D808. 10.1093/nar/gkx1011 29092050 PMC5753204

[54] Giraldo-Calderón GI, Emrich SJ, MacCallum RM, Maslen G, Dialynas E, Topalis P, et al VectorBase: an updated bioinformatics resource for invertebrate vectors and other organisms related with human diseases. Nucleic Acids Res. 2015;43: D707–13. 10.1093/nar/gku1117 25510499 PMC4383932

[55] Bottino-Rojas V, Talyuli OAC, Carrara L, Martins AJ, James AA, Oliveira PL, et al The redox-sensing gene Nrf2 affects intestinal homeostasis, insecticide resistance, and Zika virus susceptibility in the mosquito Aedes aegypti. J Biol Chem. 2018;293: 9053–9063. 10.1074/jbc.RA117.001589 29685890 PMC5995510

[56] Toubiana M, Rosani U, Giambelluca S, Cammarata M, Gerdol M, Pallavicini A, et al Toll signal transduction pathway in bivalves: complete cds of intermediate elements and related gene transcription levels in hemocytes of immune stimulated *Mytilus galloprovincialis*. Dev Comp Immunol. 2014;45: 300–312. 10.1016/j.dci.2014.03.021 24709052

[57] Schmittgen TD, Livak KJ. Analyzing real-time PCR data by the comparative CT method. Nat Protoc. 2008;3: 1101–1108. 10.1038/nprot.2008.73 18546601

[58] Livak KJ, Schmittgen TD. Analysis of relative gene expression data using real-time quantitative PCR and the 2(-Delta Delta C(T)) Method. Methods. 2001;25: 402–408. 10.1006/meth.2001.1262 11846609

[59] Zumaya-Estrada FA, Martínez-Barnetche J, Lavore A, Rivera-Pomar R, Rodríguez MH. Comparative genomics analysis of triatomines reveals common first line and inducible immunity-related genes and the absence of Imd canonical components among hemimetabolous arthropods. Parasit Vectors. 2018;11: 48 10.1186/s13071-017-2561-2 29357911 PMC5778769

[60] Armisen D, Rajakumar R, Friedrich M, Benoit JB, Robertson HM, Panfilio KA, et al The genome of the water strider *Gerris buenoi* reveals expansions of gene repertoires associated with adaptations to life on the water. BioRxiv. 2018; 10.1101/242230 PMC6249893 30463532

[61] Stanke M, Morgenstern B. AUGUSTUS: a web server for gene prediction in eukaryotes that allows user-defined constraints. Nucleic Acids Res. 2005;33: W465–7. 10.1093/nar/gki458 15980513 PMC1160219

[62] Campbell MS, Holt C, Moore B, Yandell M. Genome Annotation and Curation Using MAKER and MAKER-P. Curr Protoc Bioinformatics. 2014;48: 4.11.1–39. 10.1002/0471250953.bi0411s48 25501943 PMC4286374

[63] Oliva Chávez AS, Shaw DK, Munderloh UG, Pedra JHF. Tick humoral responses: marching to the beat of a different drummer. Front Microbiol. 2017;8: 223 10.3389/fmicb.2017.00223 28261180 PMC5306392

[64] Panfilio KA, Vargas Jentzsch IM, Benoit JB, Erezyilmaz D, Suzuki Y, Colella S, et al Molecular evolutionary trends and feeding ecology diversification in the Hemiptera, anchored by the milkweed bug genome. BioRxiv. 2017; 10.1101/201731 PMC6444547 30935422

[65] Saha S, Hosmani PS, Villalobos-Ayala K, Miller S, Shippy T, Rosendale A, et al Improved annotation of the insect vector of Citrus greening disease: Biocuration by a diverse genomics community. BioRxiv. 2017; 10.1101/099168 PMC5502364 29220441

[66] Arp AP, Hunter WB, Pelz-Stelinski KS. Annotation of the asian citrus psyllid genome reveals a reduced innate immune system. Front Physiol. 2016;7: 570 10.3389/fphys.2016.00570 27965582 PMC5126049

[67] Ioannidis P, Lu Y, Kumar N, Creasy T, Daugherty S, Chibucos MC, et al Rapid transcriptome sequencing of an invasive pest, the brown marmorated stink bug *Halyomorpha halys*. BMC Genomics. 2014;15: 738 10.1186/1471-2164-15-738 25168586 PMC4174608

[68] Sloan DB, Nakabachi A, Richards S, Qu J, Murali SC, Gibbs RA, et al Parallel histories of horizontal gene transfer facilitated extreme reduction of endosymbiont genomes in sap-feeding insects. Mol Biol Evol. 2014;31: 857–871. 10.1093/molbev/msu004 24398322 PMC3969561

[69] Rao Q, Rollat-Farnier P-A, Zhu D-T, Santos-Garcia D, Silva FJ, Moya A, et al Genome reduction and potential metabolic complementation of the dual endosymbionts in the whitefly *Bemisia tabaci*. BMC Genomics. 2015;16: 226 10.1186/s12864-015-1379-6 25887812 PMC4438442

[70] Song N, Liang A-P, Bu C-P. A molecular phylogeny of Hemiptera inferred from mitochondrial genome sequences. PLoS One. 2012;7: e48778 10.1371/journal.pone.0048778 23144967 PMC3493603

[71] Behrman EL, Howick VM, Kapun M, Staubach F, Bergland AO, Petrov DA, et al Rapid seasonal evolution in innate immunity of wild *Drosophila melanogaster*. Proc Biol Sci. 2018;285 10.1098/rspb.2017.2599 29321302 PMC5784205

[72] McTaggart SJ, Obbard DJ, Conlon C, Little TJ. Immune genes undergo more adaptive evolution than non-immune system genes in *Daphnia pulex*. BMC Evol Biol. 2012;12: 63 10.1186/1471-2148-12-63 22577801 PMC3457901

[73] Lai AG, Aboobaker A. The innate immune systems of malacostracan crustaceans exhibit both conserved and evolutionarily distinct components. BioRxiv. 2016; 10.1101/091835

[74] Obbard DJ, Welch JJ, Kim K-W, Jiggins FM. Quantifying adaptive evolution in the *Drosophila* immune system. PLoS Genet. 2009;5: e1000698 10.1371/journal.pgen.1000698 19851448 PMC2759075

[75] Han M, Qin S, Song X, Li Y, Jin P, Chen L, et al Evolutionary rate patterns of genes involved in the *Drosophila* Toll and Imd signaling pathway. BMC Evol Biol. 2013;13: 245 10.1186/1471-2148-13-245 24209511 PMC3826850

[76] Sackton TB, Lazzaro BP, Schlenke TA, Evans JD, Hultmark D, Clark AG. Dynamic evolution of the innate immune system in *Drosophila*. Nat Genet. 2007;39: 1461–1468. 10.1038/ng.2007.60 17987029

[77] Bulmer MS, Crozier RH. Variation in positive selection in termite GNBPs and Relish. Mol Biol Evol. 2006;23: 317–326. 10.1093/molbev/msj037 16221893

[78] Begun DJ, Whitley P. Adaptive evolution of relish, a Drosophila NF-kappaB/IkappaB protein. Genetics. 2000;154: 1231–1238. 10757765 10.1093/genetics/154.3.1231 PMC1460974

[79] Gordon ERL, McFrederick Q, Weirauch C. Phylogenetic evidence for ancient and persistent environmental symbiont reacquisition in largidae (hemiptera: heteroptera). Appl Environ Microbiol. 2016;82: 7123–7133. 10.1128/AEM.02114-16 27694238 PMC5118923

[80] Toenshoff ER, Gruber D, Horn M. Co-evolution and symbiont replacement shaped the symbiosis between adelgids (Hemiptera: Adelgidae) and their bacterial symbionts. Environ Microbiol. 2012;14: 1284–1295. 10.1111/j.1462-2920.2012.02712.x 22364314

[81] Kuechler SM, Dettner K, Kehl S. Characterization of an obligate intracellular bacterium in the midgut epithelium of the bulrush bug *Chilacis typhae* (Heteroptera, Lygaeidae, Artheneinae). Appl Environ Microbiol. 2011;77: 2869–2876. 10.1128/AEM.02983-10 21378044 PMC3126425

[82] Gerardo NM, Altincicek B, Anselme C, Atamian H, Barribeau SM, de Vos M, et al Immunity and other defenses in pea aphids, *Acyrthosiphon pisum*. Genome Biol. 2010;11: R21 10.1186/gb-2010-11-2-r21 20178569 PMC2872881

[83] Díaz-Albiter HM, Ferreira TN, Costa SG, Rivas GB, Gumiel M, Cavalcante DR, et al Everybody loves sugar: first report of plant feeding in triatomines. Parasit Vectors. 2016;9: 114 10.1186/s13071-016-1401-0 26928036 PMC4772290

[84] Alves CL, Araujo RN, Gontijo NF, Pereira MH. Importance and physiological effects of hemolymphagy in triatomines (Hemiptera: Reduviidae). J Med Entomol. 2011;48: 372–381. 10.1603/ME10151 21485376

[85] Noireau F, Diosque P, Jansen AM. *Trypanosoma cruzi*: adaptation to its vectors and its hosts. Vet Res. 2009;40: 26 10.1051/vetres/2009009 19250627 PMC2695024

[86] Wang J, Wu Y, Yang G, Aksoy S. Interactions between mutualist Wigglesworthia and tsetse peptidoglycan recognition protein (PGRP-LB) influence trypanosome transmission. Proc Natl Acad Sci USA. 2009;106: 12133–12138. 10.1073/pnas.0901226106 19587241 PMC2715537

[87] Anselme C, Vallier A, Balmand S, Fauvarque M-O, Heddi A. Host PGRP gene expression and bacterial release in endosymbiosis of the weevil *Sitophilus zeamais*. Appl Environ Microbiol. 2006;72: 6766–6772. 10.1128/AEM.00942-06 17021229 PMC1610295

[88] Zhang R, Zhu Y, Pang X, Xiao X, Zhang R, Cheng G. Regulation of Antimicrobial Peptides in *Aedes aegypti* Aag2 Cells. Front Cell Infect Microbiol. 2017;7: 22 10.3389/fcimb.2017.00022 28217557 PMC5291090

[89] Tanji T, Yun E-Y, Ip YT. Heterodimers of NF-kappaB transcription factors DIF and Relish regulate antimicrobial peptide genes in *Drosophila*. Proc Natl Acad Sci USA. 2010;107: 14715–14720. 10.1073/pnas.1009473107 20679214 PMC2930453

[90] Zhong X, Rao X-J, Yi H-Y, Lin X-Y, Huang X-H, Yu X-Q. Co-expression of Dorsal and Rel2 Negatively Regulates Antimicrobial Peptide Expression in the Tobacco Hornworm *Manduca sexta*. Sci Rep. 2016;6: 20654 10.1038/srep20654 26847920 PMC4742911

