# Supplementary material for: Rhodnius prolixus: Identification of missing components of the IMD immune signaling pathway and functional characterization of its role in eliminating bacteria
Source: PLoS One. 2019 Apr 3;14(4):e0214794. doi: 10.1371/journal.pone.0214794 (PMC6447187; doi:10.1371/journal.pone.0214794)
Supplement: S1 Table — The resulting sequences are available in GenBank under accession numbers beginning with MH. (DOCX) [file pone.0214794.s003.docx]

**Supplementary table 1.** *Rhodnius prolixus* IMD-pathway homologs with genes reported in GenBank and Vector Base.

| Gene | Vector Base Accession Code | GenBank accession number | Primer F 5’-3’  Primer R 5’-3’ | |  |
| --- | --- | --- | --- | --- | --- |
| rpFADD | RPRC013858 | GECK01061114.1  MH484617 | GTTTGGGCGCAATATGTGGT  AGAGCGTTGATCAGTCGACC | |  |
| rpDREDD |  | GECK01002741.1 | No direct PCR was done | |  |
| rpCaspar | RPRC001459 | GECK01051516.1  MH484616 | CTGTGGCCCAAGTGAAACG  CTCTTTTGCTGGCTTCATGC | |  |
| rpIKKβ |  | GECK01053880.1 | No direct PCR was done | |  |
| rpIAP2 | RPRC007068 | GECK01058463.1  MH484621 | GTCGCCACGTACCAACATCTG  GTTCCAACTTGAAGGGCAGCA | |  |
| rpEffete | RPRC005317 | GECK01025104.1  MH484619 | GAAGAGATCCACCAGCCCAA  GAAATATGCGAGAGTGTGAATTGT | |  |
| rpUev1a | RPRC011375 | GECK01033612.1  MH484620 | AGTTTCTCGTGACCCTTCGG  CAATCGGCGTAACTCCTGC |  | |
| rpBendless | RPRC011790 | GECK01105599.1  MH484618 | GTTTCTCTACCTCGCCGAAT  AACTGTCCATTGCATAGCGG | |  |
| rpCYLD |  | GECK01020525.1 | No direct PCR was done | |  |
