# Supplementary material for: Rhodnius prolixus: Identification of missing components of the IMD immune signaling pathway and functional characterization of its role in eliminating bacteria
Source: PLoS One. 2019 Apr 3;14(4):e0214794. doi: 10.1371/journal.pone.0214794 (PMC6447187; doi:10.1371/journal.pone.0214794)
Supplement: S2 Table — Each gene was first PCR amplified with primers in bold. These PCR amplicons then were used as template for a second PCR with primers that included the 5’ T7 promoter (complete sequence). PCR amplification profiles and purification procedures are described in the Methods section. (DOCX) [file pone.0214794.s004.docx]

**Supplementary table 2. Primers for the generation of dsRNA for use in silencing assays.**

| Gene | Primer F 5’-3’  Primer R 5’-3’ | length |
| --- | --- | --- |
| Aintegumenta | GTAATACGACTCACTATAGGGCGAATTG**GGTGGAGGATTTCTTTGGGACC**  GTAATACGACTCACTATAGGGCGAATTG**ACGCCTCGGTATTGAGAAGTTCG** | 516 |
| relish | GGATCCTAATACGACTCACTATAGGGAG**GAAGCACCCATGGACAACA**  GGATCCTAATACGACTCACTATAGGGAG**GCAAGATCAGCGCCAAATG** | 565 |
