# Supplementary material for: Rhodnius prolixus: Identification of missing components of the IMD immune signaling pathway and functional characterization of its role in eliminating bacteria
Source: PLoS One. 2019 Apr 3;14(4):e0214794. doi: 10.1371/journal.pone.0214794 (PMC6447187; doi:10.1371/journal.pone.0214794)
Supplement: S3 Table — Primers were designed using the PRIMER3, Beacon DesignerTM, and mFOLD. (DOCX) [file pone.0214794.s005.docx]

**Supplementary table 3. Primers for quantitative real time PCR**

| Gene | Primer F 5’-3’  Primer R 5’-3’ | Length | Efficiency | Source |
| --- | --- | --- | --- | --- |
| Defensin-A | GAATACTCCACTCAACCGCAAC  agggcatcatctagttgttgatgagtg | 131 | 0.99 | (30) |
| Defensin-C | CAGTACAGTCCTAATACCTAGCC  tgggcatcatctaattgatgttgagaa | 136 | 0.98 | (30) |
| Prolixicin | ACAATTTTGGTGGTGGTTGTC  GCTTGAGCTCTGGTCCTTCC | 194 | 1.00 | (31) |
| Lysozyme-B | CGATTGTGAACTGGCAAATG  TGGTGTTCAGTGAGCTTT | 183 | 0.91 | Designed here |
| α-tubulin | TTTCCTCGATCACTGCTTCC CGGAAATAACTGGGGCATAA | 129 | 0.92 | (87) |
| Relish | CCTTGTTGCCGAAAGTGAAG CTACTTGACCACATGGACCC | 216 | 0.94 | Designed here |
| Caspar | GGGCTGGGATTTGACTAACAC  GCTCGGTGACAGTGATCTTG | 109 | 1.01 | Designed here |
